# Preferential accumulation of regulatory T cells with highly immunosuppressive characteristics in breast tumor microenvironment

**DOI:** 10.18632/oncotarget.16565

**Published:** 2017-03-25

**Authors:** Azharuddin Sajid Syed Khaja, Salman M. Toor, Haytham El Salhat, Issam Faour, Navid Ul Haq, Bassam R. Ali, Eyad Elkord

**Affiliations:** ^1^ Cancer Research Center, Qatar Biomedical Research Institute, College of Science and Engineering, Hamad Bin Khalifa University, Qatar Foundation, Doha, Qatar; ^2^ Department of Medical Microbiology and Immunology, College of Medicine and Health Sciences, United Arab Emirates University, Al Ain, United Arab Emirates; ^3^ Oncology Department, Al Noor Hospital, Abu Dhabi, United Arab Emirates; ^4^ Surgery Department, Tawam Hospital, Al Ain, United Arab Emirates; ^5^ Pathology Department, Tawam Hospital, Al Ain, United Arab Emirates; ^6^ Department of Pathology, College of Medicine and Health Sciences, United Arab Emirates University, Al Ain, United Arab Emirates; ^7^ Institute of Cancer Sciences, University of Manchester, Manchester, United Kingdom

**Keywords:** regulatory T cells, FoxP3, helios, immune checkpoint receptors, primary breast cancer

## Abstract

Immunosuppressive cells such as regulatory T cells (Tregs) have an ambiguous role in breast cancer prognosis, with studies reporting both positive and negative correlations between Treg infiltration and prognosis. This discrepancy could be due to the different immunosuppressive molecules present in these cells. In the present study, we phenotypically characterize different Treg subsets infiltrating the tumor microenvironment (TME), compared to adjacent normal tissue and peripheral blood of primary breast cancer (PBC) patients. We report that the majority of tumor-infiltrating CD4^+^ and CD8^+^ T cells have terminally exhaustive phenotype as assessed by CD39 and PD-1 expressions. We also show that Tregs are accumulated in breast TME compared to normal tissue. Further characterization of Tregs showed that these are mainly FoxP3^+^Helios^+^ and express high levels of CTLA-4 and PD-1. This preferential accumulation of FoxP3^+^Helios^+^ Treg subset with co-expression of different immune inhibitory molecules might have a negative effect on breast cancer prognosis. Taken together, our results suggest that breast tumor cells might utilize Tregs, and different suppressive pathways involving CD39, PD-1 and CTLA-4 molecules in creating an immune-subversive environment for them to survive, and a dual blockade of these immunosuppressive molecules might be considered as an effective method in breast cancer treatment.

## INTRODUCTION

Immunosuppressive cells including regulatory T cells (Tregs) are known to play beneficial roles in immune homeostasis and in preventing autoimmunity. CD4^+^ Tregs are characterized by the high expression of IL-2 receptor α chain (CD25) and FoxP3 transcription factor, which is critical for their development and function [1]. Tregs exert their immunosuppressive actions through various contact-dependent and independent mechanisms that are not fully understood, but require different molecules and cytokines such as Helios, immune checkpoint receptors (ICRs; such as program death 1, PD-1 and cytotoxic T-lymphocyte-associated antigen 4, CTLA-4), TGF-β, IL-10 and IL-35 among others [2, 3]. Helios was shown to be a critical regulator of stable Tregs’ suppressive activity [4] and it is a marker of activated Tregs expressing immunosuppressive molecules GARP/LAP [5]. Tregs constitutively express CTLA-4, which is important for their function [6]. Additionally, CTLA-4 blockade impairs Treg suppressive functions [7, 8]. In cancers, Tregs hamper tumor-specific immune responses by suppressing the proliferation and activation of effector T cells and hence help in tumor evasion [9]. Several studies have reported increased levels of circulating Tregs in patients with colorectal [10], gastric [11], esophageal [11], renal cell carcinoma [12], hepatocellular [13], pancreatic and breast [14, 15] cancers. Increased levels of Tregs are also reported in the tumor microenvironment (TME) of different cancers including breast [15], colon [10] and pancreatic cancers [14].

Breast cancer is one of the most common female cancers and its incidence is increasing in the developed countries each year [16]. Despite the recent advances in screening and treatment, it remains one of the leading causes of cancer-related deaths among women worldwide. One of the reasons for the unsuccessful treatment could be due to the inability of the host immune system to mount sufficient tumor-specific immune responses [17]. Breast TME consists of different types of immune cells and the composition of these immune cell infiltrates has been reported to influence the outcome of the disease in different ways [18]. For example, a high CD8^+^ T-lymphocyte infiltration had a favorable effect on patients’ survival [19], whereas accumulation of Tregs in the TME was associated with decreased overall survival [20, 21]. On the other hand, Mahmoud et al. did not find a dominant role of FoxP3^+^ cells in breast cancer prognosis in multivariate analyses [22], while Ladoire et al. found FoxP3 expression to be associated with better survival in HER2-overexpressing breast cancer patients treated with neoadjuvant chemotherapy [23]. These discrepancies can be attributed to the method used for FoxP3 detection or the interactions between different immunosuppressive molecules expressed by Tregs. Therefore, studies investigating the phenotypic characteristics of Tregs in breast cancers are warranted.

In this study we investigated the levels and phenotypes of different immune cells infiltrating primary breast cancer (PBC) tissue and compared them with adjacent non-cancerous normal tissue (NT) and peripheral blood from the same patients. The presence of different ICRs (PD-1 and CTLA-4) on intratumoral CD4^+^ T cells and in different FoxP3/Helios Treg subsets was also investigated. We observed preferential accumulation of CD4^+^FoxP3^+^ Tregs in the TME and their levels were expanded in peripheral blood of these patients, compared with healthy donors (HD). There was a strong positive correlation between FoxP3 and Helios expressions in the TME, and CD4^+^ Tregs co-expressing FoxP3 and Helios were expanded in tumor tissue compared with normal tissue and peripheral blood. Additionally, we observed increased relative frequency of PD-1/CTLA-4 co-expressing cells within FoxP3^+^Helios^+^ and FoxP3^+^Helios^−^ Treg subsets, whereas the absolute percentages were significantly higher in FoxP3^+^Helios^+^ Treg subset, indicating the potent immunosuppressive potentials of the CD4^+^FoxP3^+^Helios^+^PD-1^+^CTLA-4^+^ Treg subset.

## RESULTS

### Accumulation of T cells in tumor-infiltrating leukocytes with exhaustive phenotype in breast cancer tissue

**Figure 1:**
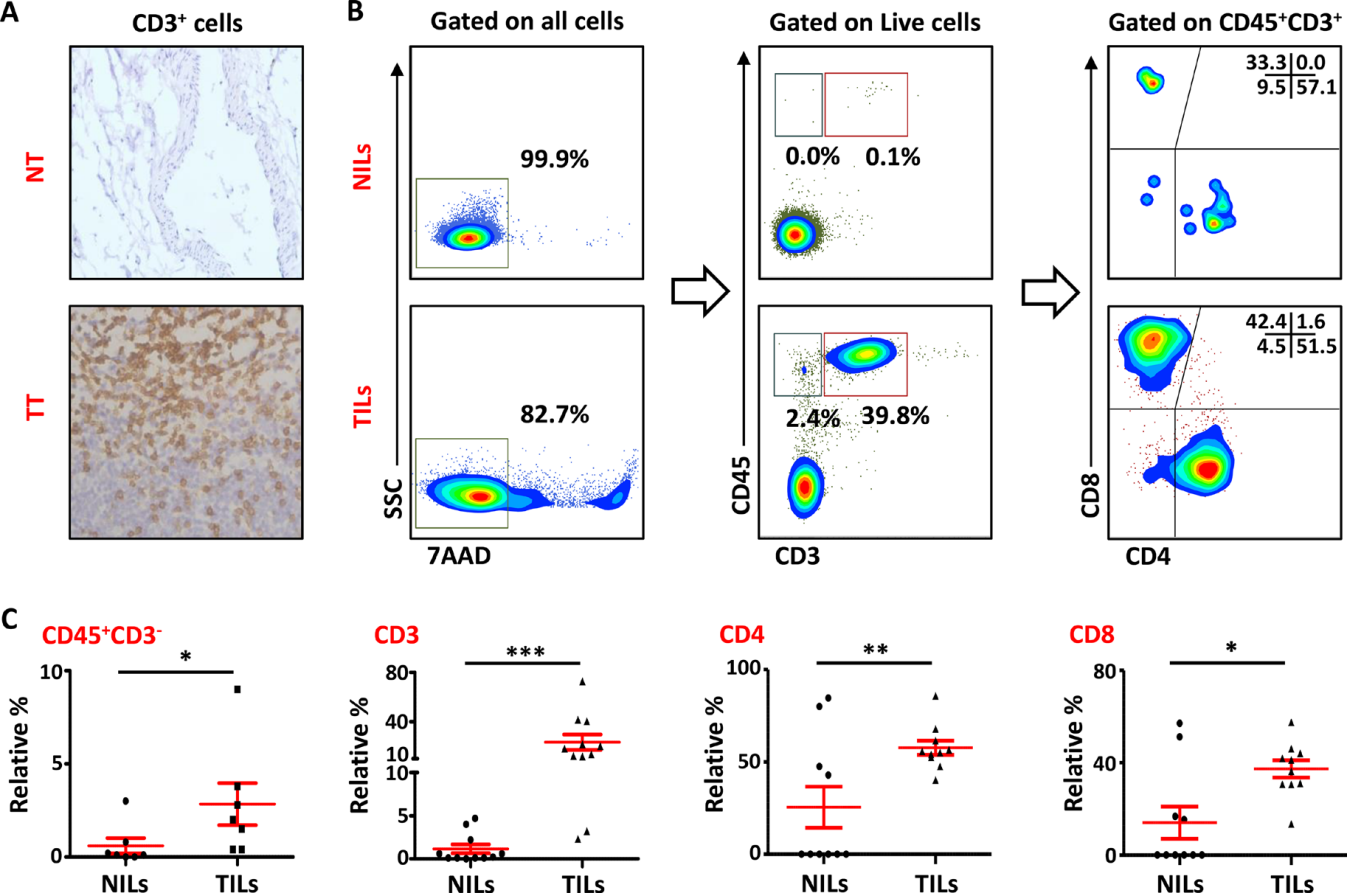
T-cell infiltration in normal and tumor tissues in primary breast cancer (**A**). Representative images of immunohistochemical staining of tumor-infiltrating CD3^+^ T cells in formalin-fixed paraffin embedded breast non-tumor (NT) and tumor tissues (TT). (**B**). Freshly isolated immune cells infiltrating NT (NILs) and TT (TILs) from 11 PBC patients were stained with 7AAD, CD45, CD3, CD4 and CD8 antibodies for identification of T cells and their subsets. Representative flow cytometric plots of surface staining from one cancer patient are shown. 7AAD dye was used to gate live cells, followed by lymphocyte identification by CD45 and CD3 stainings. Different subsets of T cells were then characterized using CD4 and CD8 antibodies. (**C**). Scatter plots showing the differences in tissue-infiltrating CD45^+^CD3^−^, CD45^+^CD3^+^, CD4^+^ and CD8^+^ cells between NILs and TILs.

Leukocyte infiltration within the TME is considered as one of the hallmarks of cancer progression [24]. In the present study, we investigated the presence of lymphocytes infiltrating PBC tissue by immunohistochemistry (IHC) and multicolor flow cytometry. As detected by IHC, most of the patients showed higher infiltration of CD3^+^ cells within tumor tissue (TT) compared with NT. CD3^+^ cells were also observed in the stroma region in TT but majority of the staining was observed within the borders of the invasive tumors (Figure 1A). We also investigated the presence and phenotype of intratumoral lymphocytes by flow cytometry. To rule out the possibility of gating dead cells and false positive results, 7AAD viability dye was used. CD45 antibody was used to detect the presence of leukocytes and CD3, CD4 and CD8 stainings were used to detect different T cell subsets. As demonstrated in Figure 1B, there was a significant accumulation of CD45^+^CD3^+^ cells in TT (23.3 ± 6.3%), compared with adjacent non-cancerous breast tissue (1.2 ± 0.5%). Within CD3^+^ population, the frequency of both CD4^+^ (NT- 28.3 ± 10.4% vs. TT- 59.6 ± 4%) and CD8^+^ (NT- 16.3 ± 6.7% vs. TT- 35.3 ± 3.9%; Figure 1C) T cells increased significantly in TT compared with NT. There was also a significant accumulation of CD45^+^CD3^−^ cells in the TME (TT- 2.8 ± 1.1%) compared with non-cancerous tissue (NT- 0.6 ± 0.4%); these cells could be of myeloid origin including granulocytic myeloid-derived suppressor cell(s) (G-MDSC) and neutrophils, which suppress host immune responses against cancer and hence promote cancer progression.

**Figure 2:**
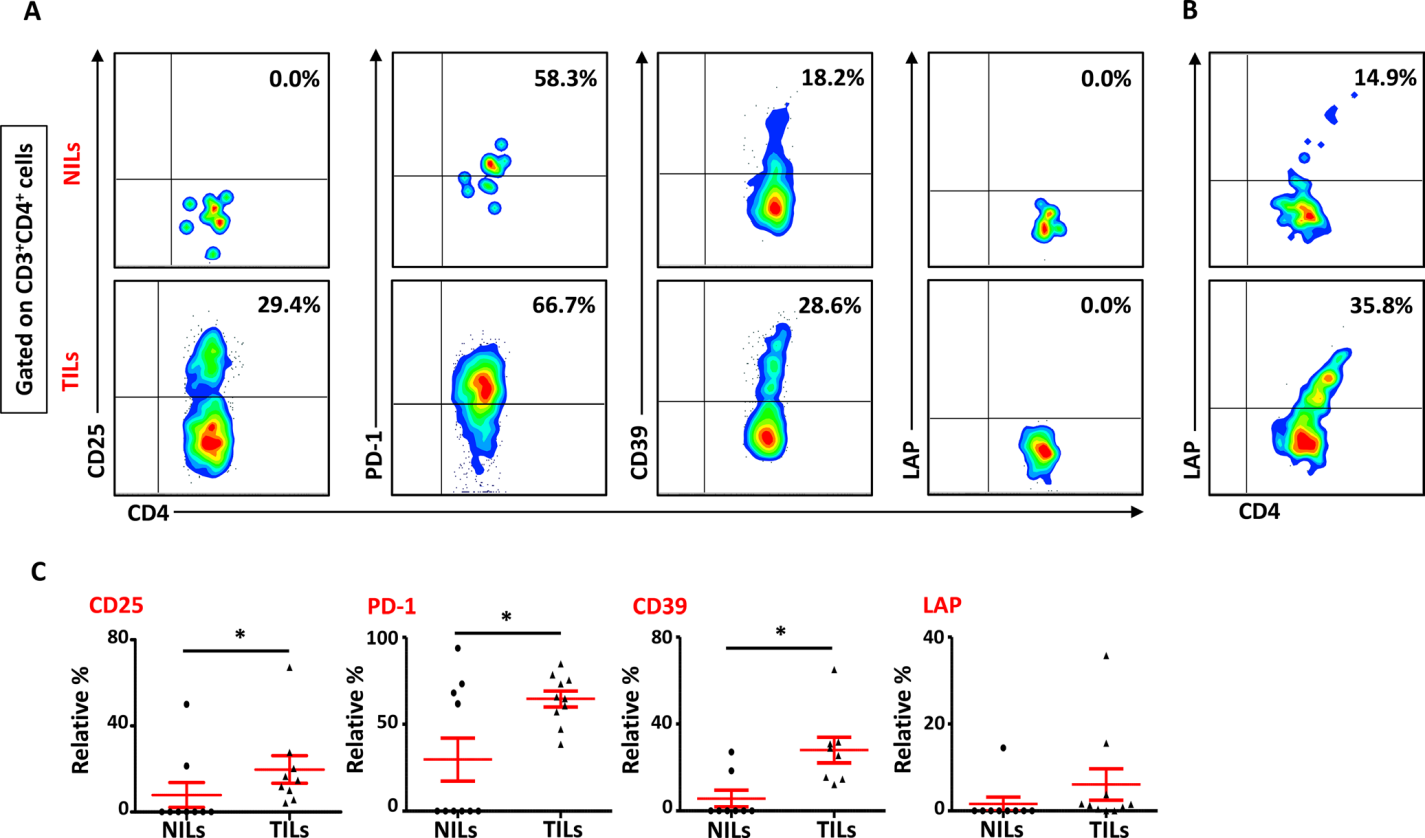
Phenotypic characteristics of CD4^+^ T cells in NILs and TILs Freshly isolated NILs and TILs were stained for CD3, CD4, CD25, PD-1, CD39 and LAP surface markers and their relative frequencies were calculated in CD4^+^ T cells. (**A**). Representative flow cytometric plots for these markers in NILs and TILs from a cancer patient. (**B**). NILs and TILs from one cancer patient showed exceptionally high levels of LAP expression. (**C**). Scatter plots showing the differences in CD25, PD-1, CD39 and LAP between NILs and TILs.

**Figure 3:**
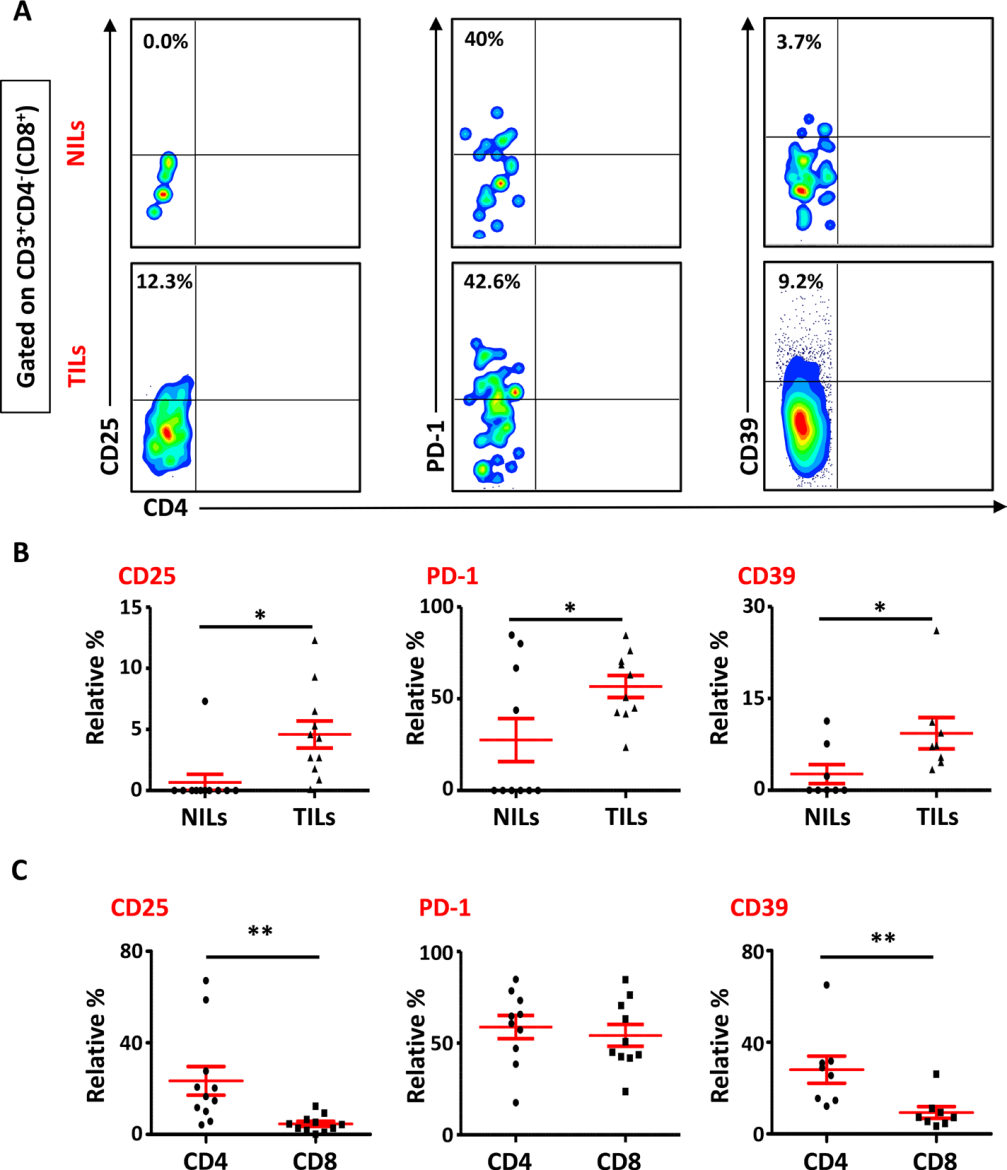
Phenotypic characterization of CD3^+^CD4^−^ (CD8^+^) T cells in NILs and TILs Freshly isolated NILs and TILs were stained for CD3, CD4, CD25, PD-1 and CD39 surface markers. Live cells were gated using 7AAD dye. Levels of CD25, PD-1 and CD39 markers were calculated in CD8^+^ T cells. (**A**). Representative flow cytometric plots for these markers in NILs and TILs from a cancer patient are shown. (**B**). Scatter plots comparing the differences between these surface markers in CD8^+^ T cells between NILs and TILs. (**C**). Scatter plots showing the differences in these markers between CD4^+^ and CD8^+^ populations in TILs.

**Figure 4:**
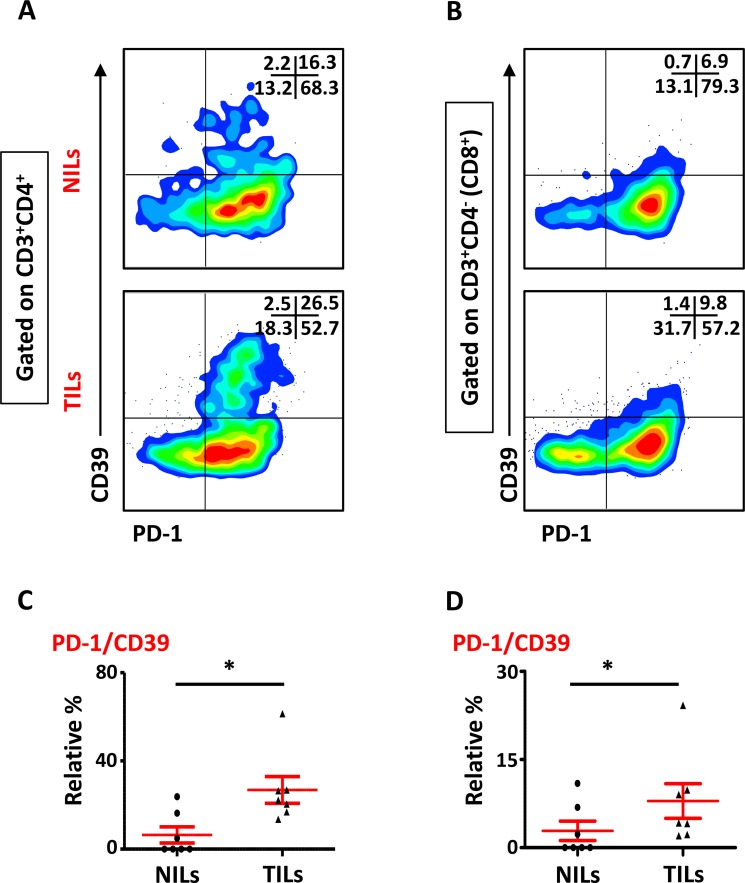
Expression of PD-1/CD39 in CD4^+^ and CD8^+^ T cells in NILs and TILs Representative flow cytometric plots for co-expression of PD-1/CD39 in CD4^+^ (**A**) and CD8^+^ (**B**) T cells. Scatter plots comparing PD-1/CD39 co-expression between NILs and TILs in CD4^+^ (**C**) and CD8^+^ (**D**) T cells.

We also studied the phenotypical characteristics of these CD4^+^ T cells infiltrating TT and NT, and found that intratumoral CD4^+^ T cells expressed higher levels of CD25 (NT- 8.5 ± 5.9% vs. TT- 21.6 ± 6.1%), PD-1 (NT- 20.8 ± 10.5% vs. TT- 57.0 ± 6.6%) and CD39 (NT- 8.2 ± 5.9% vs. TT- 28.7 ± 5.8%) compared with NT (Figure 2A and 2C). Expression of LAP (Latency-Associated Peptide) on these cells in non-activated state was also evaluated. One of the breast cancer patients in the present cohort had exceptionally high levels of LAP in CD4^+^ T cells (Figure 2B), but overall there was no significant difference in LAP expression between NILs (1.7 ± 1.4%) and TILs (4.5 ± 3.5%; Figure 2A and 2C). CD8^+^ T cells in TILs also expressed higher levels of CD25 (NT- 0.7 ± 0.7% vs. TT- 4.6 ± 1.1%), PD-1 (NT- 27.5 ± 11.7% vs. TT- 56.7 ± 6.0%) and CD39 (NT- 0.4 ± 0.3% vs. TT- 9.0 ± 3.5%), compared with NILs (Figure 3A and 3B). Within TT, the levels of CD25 and CD39 expressing CD8^+^ T cells were significantly lower than CD4^+^ T cells, whereas there was no difference in PD-1 expression between CD4^+^ and CD8^+^ T cells (Figure 3C). The co-expression of PD-1 with CD39 in intratumoral CD4^+^ and CD8^+^ T cells was also evaluated. We noticed significantly increased levels of CD4^+^ and CD8^+^ T cells co-expressing PD-1 and CD39 in TILs compared with NILs (Figure 4A–4D).

### CTLA-4 and PD-1 are upregulated in CD4^+^ T cells in breast tumor tissue

**Figure 5:**
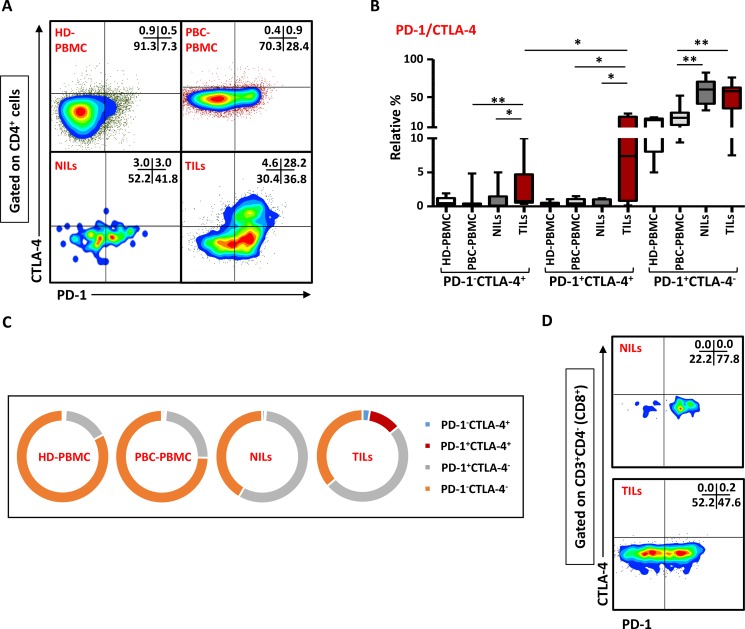
Expression of PD-1/CTLA-4 in CD4^+^ and CD8^+^ T cells PBMC from HD and PBC patients, NILs and TILs were stained for CD3, CD4 and PD-1 surface markers. After fixation and permeabilization, cells were stained for intracellular CTLA-4. Live cells were gated using Fixable Viability Dye 660. Representative flow cytometric plots showing PD-1 and CTLA-4 co-expression in CD4^+^ T cells (**A**) and whisker plots (**B**) showing differences in their expression in HD-PBMC, PBC-PBMC, NILs and TILs. (**C**). Pie charts show the relative percentages of PD-1 and CTLA-4 co-expression in CD4^+^ T cells. (**D**). Representative flow cytometric plots for co-expression of PD-1/CTLA-4 in CD8^+^ cells in NILs and TILs are shown.

We have recently observed that intratumoral CD4^+^ T cells in colorectal cancer (CRC) patients have high co-expression of ICRs including PD-1 and CTLA-4 (Syed Khaja et al. manuscript submitted). To find out if their levels are also elevated in PBC, we examined the co-expression of PD-1/CTLA-4 in CD4^+^ T cells in the present patient cohort. Representative flow cytometric plots of PD-1 and CTLA-4 co-expression in CD4^+^ T cells in PBMC of HD and PBC, NILs and TILs are shown in Figure 5A. There were no significant differences between the levels of CD4^+^ T cells co-expressing PD-1 and CTLA-4 in PBMC from HD (0.4 ± 0.1%) and PBC patients (0.7 ± 0.2%), but the frequency of CD4^+^ T cells co-expressing CTLA-4 and PD-1 increased significantly in TILs (11.5 ± 3.8%) compared with NILs (0.4 ± 0.2%) or peripheral blood (Figure 5B and 5C). Moreover, the levels of CD4^+^PD-1^−^CTLA-4^+^ T cells in TILs (2.6 ± 1.1%) were also significantly increased, compared with NILs (0.9 ± 0.6%) or peripheral blood (0.8 ± 0.5%). Within TT and NT, CD4^+^ T cells expressing PD-1 but not CTLA-4 significantly increased (TT- 49.7 ± 6.9%, NT- 57.0 ± 6.9%) compared with their levels in peripheral blood (23.9 ± 4.3%; Figure 5B). In peripheral blood of HD and PBC patients, the majority of cells did not express PD-1 or CTLA-4 and very few proportion of cells expressed only PD-1 but not CTLA-4 (Figure 5C), whereas in NILs and TILs, PD-1 expressing CD4^+^ T cells increased significantly. Within TT, PD-1/CTLA-4 co-expression was mainly observed in CD4^+^ T cells and their co-expression in CD8^+^ T cells was negligible (Figure 5D).

### Regulatory T cells are enriched in tumor tissue and peripheral blood of PBC patients

**Figure 6:**
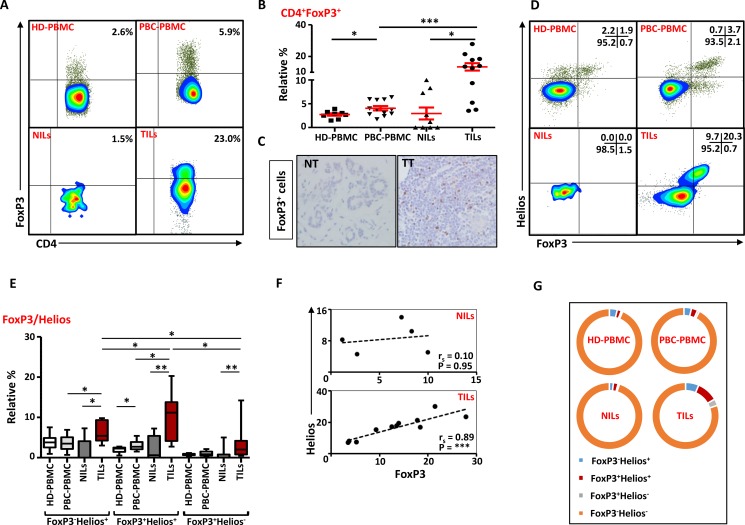
FoxP3 and Helios expression in CD4^+^ T cells PBMC from HD and PBC patients, NILs and TILs were stained for CD3 and CD4 antibodies followed by intracellular staining for FoxP3 and Helios. Live cells were first gated using Fixable Viability Dye 660. (**A**). Representative flow cytometric plots of FoxP3 staining from one cancer patient are shown. (**B**). Scatter plot showing the differences in frequencies of FoxP3^+^ Tregs between different samples. (**C**). IHC staining of FoxP3^+^ expression in one NT and TT samples. (**D**). Flow cytometric plots of FoxP3 and Helios co-expression in CD4^+^ T cells from different samples and whisker plots (**E**) showing differences in various FoxP3 and Helios-expressing Treg subsets. (**F**). Non-parametric Spearman's test showing correlations between FoxP3 and Helios expressions in NILs and TILs. (**G**). Pie charts show the relative percentages of different FoxP3 and Helios Treg subsets.

Next, we investigated the levels of Tregs within these CD4^+^ T cells infiltrating breast tumors. Accumulating evidence suggests that CD4^+^FoxP3^+^ Tregs are expanded in various cancers including breast and suppress anti-tumor immunity. In line with previous studies [15, 21, 25], we observed significant accumulation of CD4^+^FoxP3^+^ Tregs in breast tumors (13.4 ± 2.3%), compared with NT (2.9 ± 1.3%; Figure 6A and 6B). We also confirmed this finding by IHC and observed significant infiltration of FoxP3^+^-expressing cells in TT compared with NT (Figure 6C). There was also a significant accumulation of Tregs within TME compared with matching peripheral blood samples (4.1 ± 0.9%; Figure 6A and 6B). As shown in previous studies [26], PBC patients had significantly higher levels of Tregs in peripheral blood compared with HD (2.7 ± 0.3%). We also compared FoxP3^+^ Treg levels between different breast cancer pathophysiological parameters such as disease stage and grade but did not find any differences in their levels, which could be due to limited sample size (data not shown).

### FoxP3^+^Helios^+^ Tregs are increased in breast tumor tissues

It has recently been reported that Helios is essential for Tregs’ stable inhibitory activity [4]. We observed that CD4^+^FoxP3^+^ Tregs in TILs expressed higher levels of Helios and the relative percentages of Tregs co-expressing FoxP3 and Helios within the TME was significantly higher than NT or peripheral blood of cancer patients (Figure 6D and 6E). Interestingly, there was a strong positive correlation between FoxP3 and Helios expressions in TILs (Spearman's rank correlation coefficient (r_s_) = 0.89, *p* < 0.0001) but not NILs (r_s_ = 0.10, p = 0.95; Figure 6F). Levels of tumor-infiltrating FoxP3^+^Helios^+^ Tregs were significantly higher than FoxP3^−^Helios^+^ and FoxP3^+^Helios^−^ Treg subsets. Moreover, FoxP3^−^Helios^+^ and FoxP3^+^Helios^−^ Treg subsets were significantly higher in TILs, compared with NILs or PBMC (Figure 6E). PBC patients also showed a significant increase in the levels of FoxP3^+^Helios^+^ Tregs in peripheral blood, compared with HD (Figure 6E). The majority of CD4^+^ T cells in HD-PBMC, PBC-PBMC and NILs did not express either FoxP3 or Helios, and had very few FoxP3/Helios co-expressing cells, whereas the relative proportion of FoxP3^+^Helios^+^ Tregs was significantly higher in TILs (Figure 6G).

### Intratumoral FoxP3^+^Helios^+^ and FoxP3^+^Helios^−^ Tregs co-express CTLA-4 and PD-1

**Figure 7:**
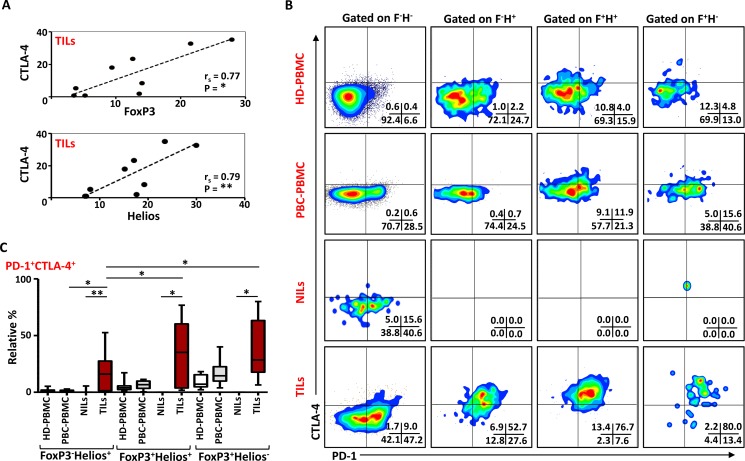
PD-1 and CTLA-4 expression in different FoxP3 and Helios Treg subsets (**A**). Non-parametric Spearman's test showing correlations between CTLA-4 and FoxP3, and CTLA-4 and Helios expressions in TILs. (**B**). Representative flow cytometric plots showing PD-1 and CTLA-4 expression in different FoxP3/Helios Treg subsets from HD-PBMC, PBC-PBMC, NILs and TILs. (**C**). Whisker plots comparing the levels of PD-1^+^CTLA4^+^ cells in FoxP3^−^Helios^+^, FoxP3^+^Helios^+^ and FoxP3^+^Helios^−^ Treg subsets within different samples.

CD4^+^FoxP3^+^ Tregs require CTLA-4 for their potent immunosuppressive activities, and CTLA-4 deficiency in Tregs impaired their suppressive functions [6]. Other studies have also defined the importance of PD-1 in Treg functions [27, 28]. In this study, we investigated any possible correlations of FoxP3 and Helios with CTLA- 4 and PD-1. There was no correlation between FoxP3 and CTLA-4 in HD PBMC (r_s_ = 0.2, *p* = 0.6), and a statistically insignificant association in PBC PBMC, (r_s_ = 0.6, *p* = 0.08). In TILs, there was a strong positive correlation between FoxP3 and CTLA-4 expressions (r_s_ = 0.77, *p* = 0.015; Figure 7A). We also observed a strong positive correlation between Helios and CTLA-4 expressions (r_s_ = 0.79, *p* = 0.01) (Figure 7A). In contrast there was an inverse correlation between CTLA-4 and Helios expressions in PBMC from HD (r_s_ = -0.78, p = 0.017). There were no significant correlations between PD-1 or CD39 with FoxP3 or Helios in TILs, NILs and PBMC of cancer patients (data not shown).

To further characterize these intratumoral Tregs and the different ICRs they co-express compared to NILs and peripheral blood, we examined the co-expression of PD-1 and CTLA-4 in different FoxP3/Helios Treg subsets. Representative flow cytometric plots of PD-1/CTLA-4 co-expression in different groups are shown in Figure 7B. In TILs, co-expression of PD-1 and CTLA-4 was observed in FoxP3^−^Helios^+^, FoxP3^+^Helios^+^ and FoxP3^+^Helios^−^ Treg subsets, which were significantly higher compared with their levels in NILs (Figure 7B and 7C). There were no significant differences in PD-1 and CTLA-4 co-expression levels within different FoxP3/Helios Treg subsets in circulation between HD and PBC patients. Within TILs, the relative percentages of ICRs co-expression were highest in FoxP3^+^Helios^+^ and FoxP3^+^Helios^−^ Treg subset, however, when investigated using absolute percentages, FoxP3^+^Helios^+^ Treg subset had the highest levels of PD-1 and CTLA-4 co-expression followed by FoxP3^−^Helios^+^ subset (data not shown).

## DISCUSSION

The TME is usually enriched with infiltrating immune cells, which accumulate as a result of a possible host immune response against cancer [29]. In this study, we found a significant accumulation of CD3^+^ T cells within breast tumor tissues, and this increase was observed in both CD4^+^ and CD8^+^ T cell subsets. Several studies have shown that the composition of these infiltrating immune cells play essential roles in disease prognosis. A high CD8^+^ T-cell infiltration within the breast tumor usually indicates a tumor-specific immune response and thus can be associated with better outcome [19]. But, as an escape mechanism, tumors secrete many chemokines and cytokines that help in attracting immunosuppressive cells such as MDSC and Tregs, which suppress effector T cells and down-regulate their anti-tumor functions [29]. A significant increase in the levels of CD45^+^CD3^−^ cells within tumor tissue in our study also indicated the presence of cells of myeloid origin including MDSC and neutrophils that play important roles in suppressing host immune responses against cancer, and hence promote cancer progression. We have recently reported an accumulation of myeloid cells in the TME but not in peripheral blood of this PBC patients’ cohort [30].

The expanded CD4^+^ T cells within the breast tumor tissue also expressed high levels of CD25, PD-1 and CD39 compared with their levels in normal breast tissue, indicating their activated but “exhaustive” state. High expression of CD25 in these cells also indicates the presence of Tregs in TILs, and elevated PD-1 and CD39 expression might suggest that these cells are exhausted and unable to mount any tumor-specific immune response. Hilchey et al. reported elevated levels of CD4^+^CD39^+^ T cells in lymph node mononuclear cells in human follicular lymphoma and blocking CD39 activity partially restored T cell hyporesponsiveness in some patients [31]. They suggested that CD39/adenosine pathway could contribute to “T-cell anergy” in tumors. In chronic lymphocytic leukemia, levels of circulating CD4^+^CD39^+^ T cells significantly increased and correlated with advanced stage of the disease [32]. In a different setting than cancer, activated T cells with high CD39 expression were prone to apoptosis in older individuals and suggested that CD4^+^CD39^+^ effector T cells do not develop into long-lived memory cells [33]. The significant increase in intratumoral CD4^+^ T cells co-expressing PD-1/CTLA-4 in our study further supports the unresponsiveness state of these cells. CTLA-4 and PD-1 are key negative regulators of antigen-specific T cell responses [28]. These different immune inhibitory pathways are exploited by tumor cells for escaping from host immune response, and hence blocking these pathways is an important strategy in cancer immunotherapy. Preclinical studies using monoclonal antibodies against CTLA-4 and/or PD-1 have shown promising results in some cancers, but unfortunately that was achieved in a small majority [34–36]. This unresponsiveness state might be due to the presence of MDSC, and MDSC suppression by epigenetic-modulating drugs greatly increased the treatment efficacy of dual blockade in xenograft mice models including metastatic 4T1 mouse breast tumor models [37]. Elevated levels of CD4^+^ T cells co-expressing CTLA-4 and PD-1 also support the dual blockade of these immunosuppressive pathways, which could be a more effective method for treating different cancers including breast [38, 39]. Data from previous *in vivo* studies have shown that combinatorial blockade of CTLA-4 and PD-1 is an effective method for reducing tumor-specific immunosuppression [40–42].

Accumulation of CD8^+^ T cells in TT would have indicated a tumor-specific immune response as a protective function against cancer, but a high PD-1 expression in these cells, compared with NT, suggested a “T-cell anergy” state and their unresponsiveness against cancer cells [43]. CD8^+^ T cells in TME also expressed high levels of CD39 compared with NT, indicating their terminal exhaustive state [44]. Moreover, a heavy Treg accumulation within the breast tumor tissues, as observed in our study and other earlier studies [18, 45], indicated a possible mechanism utilized by tumor cells to evade host immune responses from CD8^+^ T cells. Several mechanisms could contribute to the accumulation of Tregs in the TME. Tregs are usually recruited to the tumor site by chemokines and other factors, which are secreted as a result of an interplay between tumor cells and the TME, and their infiltration/expansion can lead to the suppression of effector immune cells [46]. For example, a study found that CCR4^+^ Tregs migrate to the tumor tissue through CCL22 secreted by ovarian cancer cells [47]. Recently, a critical role of CCL1/CCR8 axis in the accumulation of FoxP3^+^ Tregs in breast the TME has also been reported [15]. Treg expansion in the TME and circulation of PBC patients could also be due to either their increased proliferation or the conversion of CD4^+^FoxP3^−^ cells into CD4^+^FoxP3^+^ cells in presence of TGF-β [48, 49].

The suppressive functions of FoxP3^+^ Tregs can be modulated by the presence of different molecules including Helios and CTLA-4. We have recently reported that Tregs co-expressing FoxP3 and Helios represent a functional subset with stronger suppressive characteristics [5], and FoxP3/Helios co-expression with GARP/LAP can be used to identify expanded Treg subsets in cancer patients [50]. In line with these data, here we report a strong positive correlation between FoxP3 and Helios and their co-expression in the majority of Tregs within the TME. Moreover, within TILs, both FoxP3 and Helios strongly correlated with CTLA-4 expression. There was also a marked increase in the relative percentage of cells co-expressing both PD-1 and CTLA-4 in FoxP3^+^Helios^+^ Treg subset, suggesting that these cells have stronger immunosuppressive potentials. Some studies have shown that Treg infiltration is associated with better prognosis in breast cancer patients [23, 51], while others have shown a reduced survival of patients with higher Treg accumulation [20, 21]. This ambiguous nature of Tregs in the prognosis of breast cancer can partly be explained by the expression of different immunosuppressive molecules and functional heterogeneity of these Tregs, especially the different cytokines they secrete [52]. In CRC, Saito et al. showed that the heterogeneity of different Treg subsets affected prognosis of the disease in different ways. CRC patients with significant accumulation of CD45RA^−^FoxP3^lo^ non-Treg subset had better prognosis, compared with those with highly suppressive CD45RA^−^FoxP3^hi^ Treg subset. Furthermore, these pro-inflammatory FoxP3^lo^ non-Tregs have lower expression of CTLA-4 and TIGIT [53], indicating that immunosuppressive molecules on T cells and Tregs affect the outcome of the disease. Moreover, differences in Treg detection method can also lead to these discrepancies. We suggest that preferential accumulation of FoxP3^+^Helios^+^ Tregs co-expressing different immune inhibitory molecules might have a negative impact on breast cancer prognosis. Taken together, our results suggest that breast tumor cells might utilize immune regulatory cells such as Treg and MDSC and different suppressive pathways involving CD39, PD-1 and CTLA-4 molecules in creating an immune-subversive environment for them to survive, and a dual blockade of these immunosuppressive molecules could be a more effective approach for treating breast cancer.

## MATERIALS AND METHODS

### Tissue samples and processing of peripheral blood

Peripheral blood from 11 PBC patients and 9 HD was collected in tubes containing heparin. Additionally, tumor tissues (TT) and adjacent non-cancerous normal tissues (NT) were obtained from PBC patients who underwent surgery at Tawam Hospital, Al Ain, UAE and Al Noor Hospital, Abu Dhabi, UAE. All patients included in the study are treatment-naive prior to surgery. Table 1 shows the clinical and pathological characteristics of all participating subjects. The study was executed under ethical approval by Al Ain Medical District Research Ethics committee, Al Ain, United Arab Emirates (Protocol no. 13/23-CRD 244/13). All patients and HD provided written informed consent prior to sample collection. All experiments were performed in accordance with relevant guidelines and regulations.

**Table 1:** Characteristic features of study populations

	HD	PBC
**Number**	9	11
**Age (median)**	27 (19–45)*	50.5 (33–65)*
TNM stage		
I		4 (4)**
II		4 (4)**
III		3 (3)**
Tumor size (cm)		2 (0.8–4.5)*
Lymph node Invasion		5
Estrogen receptor positive		8
Progesterone receptor positive		7
Triple Negative		2
Histological grade		
Well/moderate		7
Poor/undifferentiated		4

HD; Healthy Donor

PBC; primary breast cancer

*Data shown represent median (range).

**Samples taken from patients for investigating tissue-infiltrating immune cells by enzyme disaggregation.

Peripheral blood mononuclear cells(s) (PBMC) were isolated from fresh whole blood by density-gradient centrifugation using Histopaque-1077 (Cat. No. 10771, Sigma-Aldrich). PBMC were frozen in cryovials at a density of 5 million cells per 1 ml freezing media (50% fetal bovine serum (FBS), 40% RPMI 1640 media and 10% DMSO) to be used in batches for subsequent analysis.

### Immunohistochemistry

Formalin-fixed, paraffin-embedded NT and TT sections were stained for CD3 and FoxP3 markers by immunohistochemistry. Tissue sections were de-paraffinized in xylene and rehydrated with decreasing concentrations of ethanol. Antigen retrieval was performed at 90°C for 5 min using citrate buffer (pH 6.0). After serial blocking with 3% hydrogen peroxide (in methanol) and protein block (1% bovine serum albumin, 0.05% Tween 20 in PBS), tissue sections were incubated overnight at 4°C with primary monoclonal antibodies against CD3 (M7254, Dako) and FoxP3 (14-4777-82, eBioscience). The sections were then incubated at room temperature for one hour with biotinylated immunoglobulin (E0353, Dako) and another one hour with peroxidase-conjugated streptavidin (P0397, Dako) followed by the addition of DAB+ substrate chromogen (K3468, Dako) for color development. Sections were then counterstained by hematoxylin solution.

### Isolation of immune cells from breast tissues by enzyme disaggregation

Enzyme disaggregation (ED) of freshly resected breast TT and NT for immune cell isolation was performed as described previously [54]. Tissues were cut into pieces and then enzymatically digested in RPMI-1640 medium containing 1% penicillin/streptomycin and enzyme cocktail, consisting of 1mg/ml Collagenase (C0130), 100 μg/ml Hyaluronidase type V (H3506) and 30 IU/ml of Deoxyribonuclease I (D5025; all from Sigma-Aldrich) and incubated on a mixer at 37°C for 60 minutes under slow rotation. The cell suspension was then filtered through a 100 μm cell strainer (352360, BD Falcon) to remove any cellular debris and aggregates, followed by two washes with RPMI-1640 media and resuspending in complete media (RPMI-1640 media with 10% FBS and 1% penicillin/streptomycin). Cells isolated from TT (TILs, tumor-infiltrating leukocytes) and NT (NILs, non-tumor-infiltrating leukocytes) were used for flow cytometric staining.

### Multicolor flow cytometry

Cells, isolated after ED, were suspended in 100μl staining solution (PBS with 2% FBS and 0.1% sodium azide) and were incubated with human Fc receptor blocking reagent (130-059-901, Miltenyi Biotec). 7AAD viability dye (00-6993-50, eBioscience) was then added to discriminate between live and dead cells. Cells were then stained with cell surface antibodies; CD45-FITC (11-0459-42, eBioscience), CD3-APC-H7 (560176, BD Biosciences), CD4-Alexa Fluor 700 (300526, BioLegend), CD25-PE/Cy7 (356108, BioLegend), LAP-PE (349604, BioLegend), PD-1-PE/Dazzle™ 594 (329940, BioLegend), and CD39-PerCP/Cy5.5 (328218, BioLegend) for 30 minutes at 4°C. After staining, cells were washed and cell pellet was resuspended in flow cytometry staining buffer (eBioscience).

For intracellular staining, Fixable Viability Dye eFluor^®^ 660 (FVD 660; 65-0865-14, eBioscience) was added after blocking with FcR blocker (Miltenyi Biotec). Cells were then labeled with CD3-APC-H7 (BD Biosciences), CD4-Alexa Fluor 700 (BioLegend) and PD-1-PE/Dazzle^™^ 594 (BioLegend) for 30 minutes at 4°C. Following incubation, cells were washed twice with staining solution and fixed/permeabilized using fixation/permeabilization buffer (eBioscience) at 4°C for 45 min. After two washes with permeabilization wash buffer, cells were blocked using mouse serum (M5905, Sigma-Aldrich) and rat serum (R9759, Sigma-Aldrich) for 10 min and stained with CTLA-4-PerCP-eFluor^®^ 710 (46-1529-42, eBioscience), FoxP3-PE-Cy7 (25-4776-42, eBioscience) and Helios-FITC (137214, BioLegend) antibodies for another 30 minutes at 4°C. Cells were washed twice with permeabilization wash buffer (eBioscience) and resuspended in flow cytometry staining buffer. All data were acquired with a BD FACSCanto II flow cytometer using BD FACSDiva software (BD Bioscience) and analyzed on BD FACSuite software (BD Biosciences).

### Statistical analyses

All statistical analyses were performed using GraphPad Prism 5.0 software (GraphPad Software, Inc.) and Microsoft Excel (Microsoft Corporation). Shapiro-wilk normality test was performed to test if data are normally distributed, followed by paired/Wilcoxon matched-pairs signed rank test or unpaired/Mann-Whitney tests were used to examine the differences within groups or between groups, respectively. Data are represented as mean ± standard error (s.e.m). Correlations between different markers were calculated using non-parametric Spearman's correlation test. A *p value* < 0.05 was considered statistically significant. Flow cytometric plots show representative examples of the relative percentage of each population/subpopulation. For absolute or calculated percentages of a particular subpopulation, its relative percentage is multiplied with the relative percentage of its parent population and the resulting value was divided by 100 and presented as absolute percentage.

## Abbreviations

CTLA-4: cytotoxic T-lymphocyte-associated antigen 4
CRC: colorectal cancer
ED: enzyme disaggregation
HD: healthy donors
ICR: immune checkpoint receptor
IHC: immunohistochemistry
LAP: latency-associated peptide
MDSC: myeloid-derived suppressor cells
NILs: non-tumor-infiltrating leukocytes
NT: non-tumor tissue
PBC: primary breast cancer
PBMC: peripheral blood mononuclear cells
PD-1: program death 1
TILs: tumor-infiltrating leukocytes
TME: tumor microenvironment
Tregs: regulatory T cells
TT: tumor tissue
